# Kinetic studies of HIV-1 and HIV-2 envelope glycoprotein-mediated fusion

**DOI:** 10.1186/1742-4690-3-90

**Published:** 2006-12-04

**Authors:** Stephen A Gallo, Jacqueline D Reeves, Himanshu Garg, Brian Foley, Robert W Doms, Robert Blumenthal

**Affiliations:** 1 Center for Cancer Research Nanobiology Program, National Cancer Institute at Frederick, National Institutes of Health, Frederick, MD, USA; 2 Dept. Microbiology, University of Pennsylvania, Philadelphia, PA, USA; 3 Los Alamos National Laboratories, Los Alamos, NM, USA

## Abstract

**Background:**

HIV envelope glycoprotein (Env)-mediated fusion is driven by the concerted coalescence of the HIV gp41 N-helical and C-helical regions, which results in the formation of 6 helix bundles. Kinetics of HIV Env-mediated fusion is an important determinant of sensitivity to entry inhibitors and antibodies. However, the parameters that govern the HIV Env fusion cascade have yet to be fully elucidated. We address this issue by comparing the kinetics HIV-1_IIIB _Env with those mediated by HIV-2 from two strains with different affinities for CD4 and CXCR4.

**Results:**

HIV-1 and HIV-2 Env-mediated cell fusion occurred with half times of about 60 and 30 min, respectively. Binding experiments of soluble HIV gp120 proteins to CD4 and co-receptor did not correlate with the differences in kinetics of fusion mediated by the three different HIV Envs. However, escape from inhibition by reagents that block gp120-CD4 binding, CD4-induced CXCR4 binding and 6-helix bundle formation, respectively, indicated large difference between HIV-1 and HIV-2 envelope glycoproteins in their CD4-induced rates of engagement with CXCR4.

**Conclusion:**

The HIV-2 Env proteins studied here exhibited a significantly reduced window of time between the engagement of gp120 with CD4 and exposure of the CXCR4 binding site on gp120 as compared with HIV-1_IIIB _Env. The efficiency with which HIV-2 Env undergoes this CD4-induced conformational change is the major cause of the relatively rapid rate of HIV-2 Env mediated-fusion.

## Background

The origins of Human Immunodeficiency Virus (HIV) can be traced to zoonotic transmissions of Simian Immunodeficiency Virus (SIV) to humans from at least two different kinds of non-human primates [1]: HIV-1, which came from chimpanzees, and HIV-2, which came from sooty mangabeys. While similar in many ways, there are important differences between HIV-1 and HIV-2 that provide insights into virus evolution, tropism and pathogenesis [2]. Major differences include reduced pathogenicity of HIV-2 relative to HIV-1, enhanced immune control of HIV-2 infection and often some degree of CD4-independence. Despite considerable sequence and phenotypic differences between HIV-1 and 2 envelopes, structurally they are quite similar. Both membrane-anchored proteins eventually form the 6-helix bundles from the N-terminal and C-terminal regions of the ectodomain [3], which is common to many viral and cellular fusion proteins and which seems to drive fusion [4]. HIV-1_IIIB _gp41 helical regions can form more stable 6-helix bundles than HIV-2_SBL _gp41 helical regions [3,5]; however HIV-2 fusion occurs at a lower threshold temperature (25°C), does not require Ca^2+ ^in the medium, is insensitive to treatment of target cells with cytochalasin B [6], and is not affected by target membrane glycosphingolipid composition [7].

In order to elucidate mechanisms of HIV envelope glycoprotein-mediated fusion we have kinetically resolved steps in the pathway of HIV-1 membrane fusion [8]. To gain a better understanding of the molecular mechanisms underlying these steps, we compared kinetic parameters of HIV-1_IIIB _with two strains of HIV-2. We found a significant difference in fusion kinetics, which appears to be related to the CD4-induced rate of engagement of HIV gp120 with its coreceptor. Since the CD4-induced binding of gp120 proteins to CXCR4 is not very different between the different strains, we surmise that in the intact Env other regions (e.g. the cytoplasmic tail) may have a profound influence on the conformational changes in the surface-exposed portions of the envelope glycoproteins.

## Results

### Fusion kinetics

**Figure 1:**
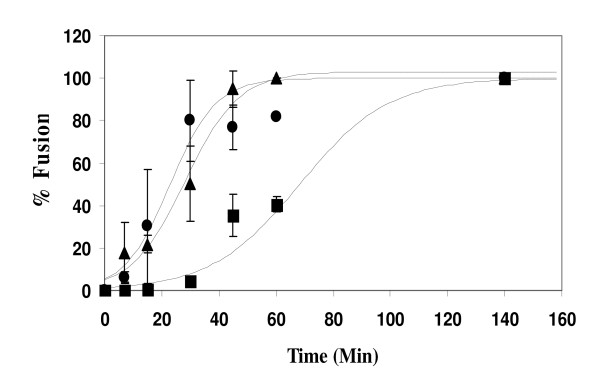
**Kinetics of HIV-1 and HIV-2 Env-mediated fusion**. HIV-1_IIIB _(squares), HIV-2_SBL _(triangles), and HIV-2_ROD _(circles) Env proteins were expressed in HeLa cells using vaccinia recombinants as described in Materials and Methods. Target SupT1 cells, labeled with calcein, were added to the plated HeLa cells, labeled with CMTMR, at various times during a two hour period at 37°C. The cells were then examined by fluorescence microscopy for dye transfer indicating cell-cell fusion. Lines represent fits to the sigmoidal equation f = *a*/(1-exp [-*b*(*t *- *t*_1/2_)]) using Sigmaplot (SPSS, Chicago). Values of time for half maximal fusion (t_1/2_) are 63 ± 6, 28 ± 2 and 23 ± 4 minutes for HIV-1_IIIB_, HIV-2_SBL _and HIV-2_ROD_, respectively.

We examined the dye transfer that occurs as result of fusion between HeLa cells infected with recombinant vaccinia viruses expressing Env proteins and labeled with a red tracker dye and target SupT1 cells labeled with calcein at different times of co-culture at 37°C. Figure 1 shows that once cells expressing HIV-1_IIIB _Env were mixed with SupT1 cells, fusion began after a lag phase at 37°C of about 30 min, with 50% of maximum fusion (t_1/2_) occurring at 63 ± 6 min. HIV-2_SBL _and HIV-2_ROD _Env-mediated fusion, on the other hand, showed no appreciable lag time and 50% of maximum fusion was reached in 23 ± 4 and 28 ± 2 minutes, respectively.

### Binding of HIV-1 and HIV-2 gp120 to CD4 and CXCR4

**Figure 2:**
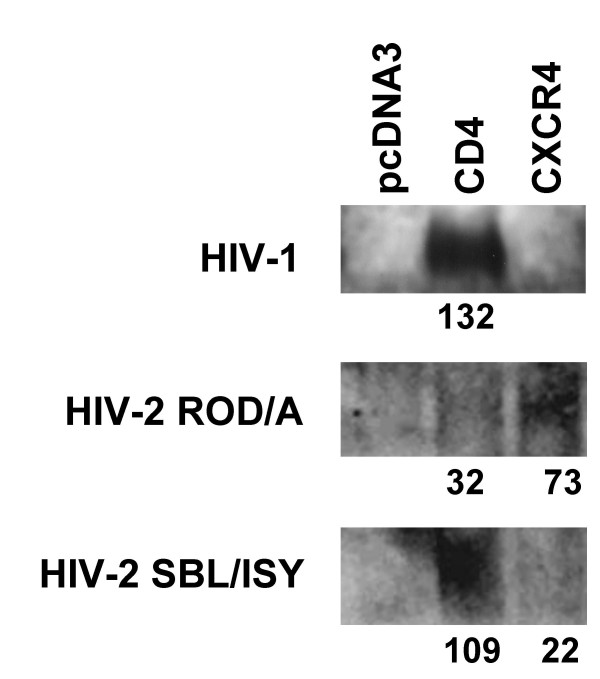
**Binding of soluble gp120 to cellular CD4 orCXCR4**. Receptor binding efficiencies of gp120s were determined using a cell surface-binding assay in which bound protein was detected by Western blot analysis as described in Materials and Methods. Binding to CXCR4 was performed in the presence of sCD4 to expose the coreceptor binding site. HIV-1_IIIB _and HIV-2_SBL _gp120 exhibited relatively high CD4 binding efficiencies but weak CXCR4 binding. HIV-2_ROD _gp120 exhibited relatively weak binding to CD4 and relatively high binding to CXCR4. Numbers indicate mean band intensity following subtraction of background pcDNA3 lane intensity.

In previous studies we have found that fusion rates can be dependent on the affinity with which an Env binds to its coreceptor [9,10]. Potentially, differences in CD4 affinity could also impact fusion kinetics. We therefore performed studies to assess the binding of soluble gp120s derived from each of the virus strains to CXCR4 or CD4. Cells expressing no receptor (pcDNA3 transfected), CD4 or CXCR4 were incubated with equivalent amounts of purified HIV-1 or HIV-2 gp120s with or without the presence of soluble CD4 (to allow CXCR4 binding), and were then washed, lysed and assayed for binding through Western blot analysis (Figure 2). HIV-2_ROD _gp120 bound CD4 poorly as compared with HIV-2_SBL _gp120, but HIV-2_ROD _gp120 exhibited stronger binding to CXCR4 as compared to HIV-2_SBL _gp120. HIV-1_IIIB _gp120 was more similar to HIV-2_SBL _gp120 in its CD4 and CXCR4 binding profile than HIV-2_ROD _gp120 (Figure 2). Specificity of binding was demonstrated by sCD4 inhibition of CD4 binding and a lack of CXCR4 binding in the absence of sCD4 (data not shown).

These binding data were further corroborated by inhibition studies of HIV-1 and HIV-2-mediated fusion. Dose-response curves were generated for inhibition of gp120-CD4 binding by Leu3A, and for gp120-CXCR4 binding by AMD3100. Table 1 shows the IC50 values derived from these curves. Higher amounts of inhibitor are required to displace ligands with high affinity for their receptor, provided that the ligands bind in a similar fashion. The high IC50 for inhibition of HIV-2_ROD _by AMD3100 (Table 1) is entirely consistent with its binding potency shown in figure 2. HIV-1_IIIB _and HIV-2_SBL_, on the other hand, had low IC50's for inhibition by AMD3100 (Table 1) consistent with low affinity for CXCR4 as suggested by the data in figure 2. The gp120-CD4 binding data shown in figure 2 are also consistent with inhibition of fusion by Leu3A. HIV-2_ROD _showed little binding to CD4, and its IC50 for inhibition by Leu3A was the lowest compared to HIV-1_IIIB _and HIV-2_SBL_. The latter two showed binding to CD4 in the Western blot assay and had higher IC50 values. The binding of HIV-1 and HIV-2 gp120 to CD4 and CXCR4 per se does therefore not appear to account for the differences in fusion kinetics.

**Table 1:** Inhibition of HIV-1 and HIV-2 Env-mediated fusion. AMD3100 or Leu3A were added to cocultures of HIV-expressing cells and target cells at the time of co-culture and fusion was measured as described in Methods. IC50 values were derived from dose-response by fitting the data to a hyperbolic decay function.

	**IC50 (μg/ml) for fusion at 37°C**	
	**AMD3100**	**Leu3A**

HIV-1_IIIB_	0.53 ± 0.07	0.3 ± 0.1
HIV-2_ROD_	>10	0.03 ± 0.02
HIV-2_SBL_	0.14 ± 0.02	0.09 ± 0.01

### Escape from inhibition by Leu3A and C34

**Figure 3:**
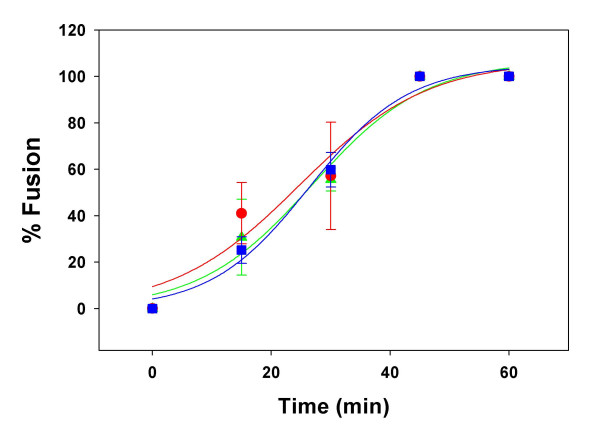
**Time course for escape from HIV-2**_SBL_**Env-mediated fusion inhibition**. Fusion was measured as described in the legend to figure 1. Leu3A (3 μg/ml, triangles), AMD3100 (40 μM, circles) and SIV C34 (2 μM, squares) were added at different times following co-culture at 37°C and residual fusion was measured following their time of addition. The curves were fitted by the sigmoidal function given in the legend to figure 1; values of time for half maximal fusion (t_1/2_) are 26.9 ± 4.1, 24.8 ± 6.4 and 26.4 ± 2.3 minutes for Leu3A (green), AMD3100 (red) and C34 (blue), respectively.

In previous studies we had dissected the kinetics of HIV-1 Env-mediated fusion by adding inhibitors that act at the various steps of the fusion reaction [11] at different times following co-culture of Env-expressing cells with target cells. In those studies we observed a large time differential between losses of sensitivity to Leu3A as compared to AMD3100. However, loss of sensitivity to C34 occurred nearly concomitantly with loss of sensitivity to AMD3100 indicating that the HIV-1 gp41 6-helix bundle formation occurs rapidly after the engagement of gp120 by CXCR4 in the HIV-1 env-mediated fusion process. In order to analyze the rate-limiting step in HIV-2_SBL _Env-mediated fusion we performed similar loss of sensitivity studies. Previously we had shown that SIV C34 is a good inhibitor of HIV-1 as well as HIV-2 fusion [3]. We used concentrations of C34 at which HIV-2_SBL _Env-mediated fusion was completely inhibited. Figure 3 shows that the kinetics of loss of sensitivity to Leu3A, AMD3100 and C34 were indistinguishable with t_1/2_'s of about 27 min, indicating that the HIV-2 envelope glycoprotein assumes its CXCR4-grabbing conformation very rapidly after engagement with CD4.

## Discussion

The current model of HIV viral entry involves the binding of the trimeric viral Env glycoprotein gp120/gp41 to cell surface receptor CD4, which triggers conformational changes in the envelope proteins. Gp120 is then re-positioned allowing gp41 to undergo conformational changes that result in the formation of the gp41 "pre-hairpin" [12-14]. Upon engagement with chemokine co-receptors CXCR4 or CCR5 [15,16], the C-terminal heptad repeat region and the leucine/isoleucine zipper region form the thermostable 6-helix bundle, which drives membrane merger and eventual fusion [17]. In the case of HIV-1_IIIB _it appears that the pre-hairpin conformation is quite a long lasting state [11,18]. Previously, we had attributed the relatively slow kinetics of HIV-1 Env-mediated fusion to the stochastic nature of HIV Env triggering giving rise to a relatively low probability of 6-helix bundle formation and fusion [8]. We had invoked the "harpoon" model [15] according to which the HIV-1 gp120 is searching to engage its co-receptor following initial conformational changes induced by CD4 binding. We had shown that HIV-1 gp41 6-helix bundle formation occurs rapidly after the engagement of gp120 by CXCR4 in the HIV-1 Env-mediated fusion process [11]. We reasoned that higher gp120-coreceptor affinities may result in more rapid gp120-coreceptor binding leading to enhanced rates of 6-helix formation and fusion. This hypothesis has been born out in studies showing that envelope:coreceptor affinity correlates with fusion kinetics [9,10].

In this study we show that fusion mediated by the HIV-1_IIIB _Env exhibits slower kinetics as compared to that of two different HIV-2 Envs (Figure 1). Since we observed little CD4-induced binding of gp120 from HIV-1_IIIB _and HIV-2_SBL _to CXCR4 under steady-state condition (figure 2), and similar AMD3100 susceptibilities (Table 1) that are in contrast to efficient CXCR4 binding and markedly reduced AMD3100 susceptibility for HIV-2_ROD_, the difference in fusion rates mediated by these HIV-1 and HIV-2 Envs are not likely due to differences in gp120-coreceptor affinity per se. However, the time of addition experiments (figure 3) indicate that the major difference in kinetics between HIV-1_IIIB _and HIV-2_SBL _lays in the efficiency of conformational changes that occur after CD4 is bound and that result in the formation of the coreceptor-binding site. In the case of HIV-1, exposure of the coreceptor binding site has not yet occurred immediately after the stage that Leu3A can block. It has been shown that binding of a single CD4 molecule to an envelope trimer leads to conformational changes in all three gp120 molecules [19]. This process may take a relatively long time in the case of HIV-1 Env. By contrast, the coreceptor binding site is rapidly exposed following CD4 binding in the case of HIV-2 Env consistent with the observation that HIV-2 strains (in contrast to HIV-1) are often able to infect cells CD4-independently. Since CD4-induced binding of soluble gp120 molecules to CXCR4 was not very different between the HIV-1_IIIB _and HIV-2_SBL _strains, we surmise that in the intact Env other regions may have a profound influence on this conformational change.

**Figure 4:**
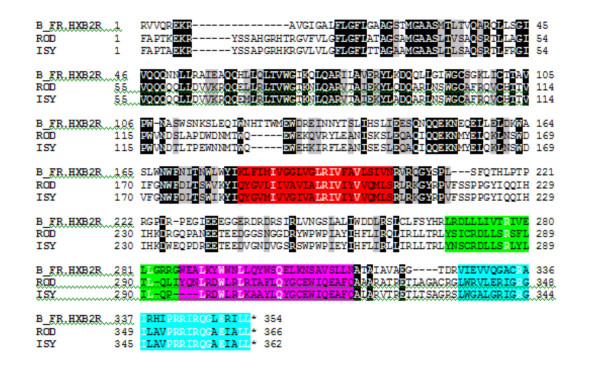
**Sequence comparison of HIV-1 and HIV-2 Envs**. The HIV-1 subtype B infectious molecular clone HXB2 Env gp41 sequence (Database accession number K03455) is aligned to the HIV-2 ROD (M15390) and ISY (J04498) Env gp41 sequences. Amino Acid sites conserved in all 3 sequences are shaded black, and those conserved in 2 of the 3 are shaded grey. Numbering is based on the HXB2 amino acid sequence. The sequences corresponding approximately to the different regions of HIV-1 gp41 have been highlighted as follows: Membrane Anchor (180–203), red; Lentivirus Lytic Peptide-3 (268–286), green; Lentivirus Lytic Peptide-2 (287–313), cyan; Lentivirus Lytic Peptide-1 (326–354), turquoise. Note the lack of homology in LLP-2 and 3 sequences, whereas LLP-1 (337–354) appears to be very homologous between the three strains.

In order to examine other regions that may affect the rate of HIV Env-mediated fusion we performed a sequence comparison between HIV-1_HxB2 _(which is similar to HIV-1_IIIB_) and the two HIV-2 strains studied in this paper (figure 4). The regions considered important for fusion (fusion peptide, N-helical region, membrane proximal region and transmembrane anchor seem to be well conserved between the strains. The C-helical regions appear to be dissimilar which could account for the differences in 6-helix bundle stabilities between HIV-1_IIIB _and HIV-2 [3,5]. However, the differences in gp41 refolding into 6-helix bundles in the intact envs do not correlate with 6-helix bundle stabilities of the peptides derived from those regions.

Another region of HIV-1 gp41 that has profound effects on fusion rates is the cytoplasmic tail [20]. HIV-1, HIV-2 and SIV CTs are remarkably long and contain domains that likely interact with host cell components, such as calmodulin [21,22], α-catenin [21], p115-RhoGEF [23], Prenylated Rab acceptor protein [24], or AP-1 clathrin adaptor proteins [25]. Three alpha helical "lentivirus lytic peptide" domains (LLP-1, LLP-2 and LLP-3) highly conserved in HIV-1 have been implicated in interacting with the cytoplasmic leaflet of plasma membrane, decreasing bilayer stability, altering membrane ionic permeability, and mediating cell killing [26-28]. Poorly defined regions of gp41 have also been implicated in interacting with viral matrix proteins during virion assembly [29,30]. This interaction also modulates Env function in that gp41 is more stably associated with immature rather than mature viral particles [30], and cleavage of the p55 Gag precursor protein by the viral protease is required to generate Envs with maximal fusogenicity [31,32].

Truncations of gp41 proximal to the most N-terminal alpha helix, LLP-2, produced a significant increase in the rate of HIV-1 Env-mediated cell fusion [20,33]. These effects were not seen with a truncation distal to this domain and before LLP1 [20]. These results were observed for X4-, R5-, and dual-tropic Envs on CXCR4- and CCR5-expressing target cells. Sequence comparisons of gp41 derived from HIV-1, HIV-2 and SIV indicate high homology between the three viral Envs in the LPP-1 region but not in the LLP-2 region of the cytoplasmic tail (the sequence comparisons can be obtained from Brian Foley upon request). In the case of certain HIV-1 Env strains it has been shown that CT truncations prior to LLP-2 profoundly affect the exposure of CD4-induced epitopes on gp120 [20,34], indicating that "inside-out" signalling changes the conformation in the Env ectodomain. Although we have performed this analysis by comparing only one HIV-1 and two HIV-2 strains we believe that the methodology developed here can be expanded to include comparisons between various strains of HIV-1 and HIV-2. Future studies that involve domain swapping between HIV-1 and HIV-2 gp41 CT's will determine whether our LLP-2 hypothesis turns out to be correct.

## Conclusion

We find that the differences in fusion mediated by HIV-1 and HIV-2 Env are due to the rates by which the Envs assume a conformation that expose the binding sites on gp120 to the CXCR4 following engagement with CD4. In spite of the fact that CD4-induced binding to CXCR4 of isolated gp120 derived from HIV-1_IIIB _and HIV-2_SBL _were similar, their rate of binding to the co-receptor was markedly different in the context of the complete Envs. We speculate that sequences in the cytoplasmic tail of the Env may have a profound influence upon the rate by which the extracytoplasmic portion of gp120 assumes its CXCR4-engageable conformation.

## Methods

### Cells

QT6, 293T and HeLa cell lines were cultured in DMEM supplemented with 10% fetal calf serum, 60 mg/ml of penicillin and 100 mg/ml streptomycin. Sup-T1 cells (Non-Hodgkin's T-cell lymphoma cell line) were cultured in RPMI supplemented with 10% serum, 60 mg/ml of penicillin and 100 mg/ml streptomycin.

### Inhibitors

The fusion inhibitor SIV C34 [3] was dissolved in PBS at a stock concentration of 500 uM. The CXCR4 antagonist AMD3100 [35], a kind gift from Anormed, Inc. (Langley, Canada), was dissolved in PBS at a stock concentration of 1 μg/ul. Leu3A, an antibody against the gp120 binding site of CD4 and a highly effective fusion inhibitor [36] (BD Biosciences, San Jose, CA), was dissolved in 0.1% Azide at 25 ug/ml. All inhibitors were stored at 4°C.

### Plasmids

The 3' half proviral clone of HIV-2_SBL _(KF-3; kindly provided by G. Franchini) was used as a template for PCR amplification of the envelope gene using 5' CACCATGAGTGGTAAAATTCAGCTGC 3' and 5' CTCCTTGCTGATATCTCTGTCCCTCA 3' oligonucleotides. The PCR product was cloned into the pcDNA3.1 D/V5-His-TOPO expression vector (Invitrogen, Carlsbad, CA) to generate an sbl/isy gp160 expression construct. A stop codon was introduced at the gp120/gp41 cleavage junction of the sbl/isy gp160 expression construct using the 'Quikchange' site directed mutagenesis kit (Stratagene, La Jolla, CA) and 5' GGGAGACATAAGAGATGAAAGCTTGTGCTAGGGTTC 3' and 5' GAACCCTAGCACAAGCTTTCATCTCTTATGTCTCCC 3' oligonucleotides to generate a gp120 expression construct. These oligonucleotides also introduce an HindIII restriction enzyme site (for screening purposes) down stream of the stop codon. HIV-2_ROD/A _and HIV-1_IIIB _gp120 expression vectors have been described previously [37,38].

### Vaccinia recombinants

HIV-1 and HIV-2 Env proteins were expressed in HeLa cells using the following vaccinia recombinants;vPE16, for the IIIB strain of HIV-1 [39], vvROD, for the ROD strain of HIV-2 [40], and vSC50 for the SBL/ISY strain of HIV-2 [41]. These recombinants were incubated with cells overnight at a ratio of 10:1 infectious virions to cells. HeLa cells were plated on 12 or 24 well plates overnight before infection (Costar, Cambridge, MA). The recombinant vTF1.1 vaccinia virus encoding T7 polymerase was used to drive expression of plasmids with the T7 promoter.

### Cell-Cell fusion assay

For dye transfer assays [11], HeLa cells infected with recombinant vaccinia viruses expressing Env proteins and labeled with CMTMR (494/517, red) and target SupT1 cells labeled with Calcein (541/565, green) in suspension were co-cultured (Molecular Probes, Eugene, OR). Kinetic experiments were conducted in which target SupT1 cells in suspension in 37°C media were added to plated effector cells at various times during a two hour period. The cells were then examined by fluorescence microscopy for dye transfer between Env and receptor expressing cells indicating cell-cell fusion. Phase and fluorescent images were collected using an Olympus IX70 coupled to a CCD camera (Princeton Instruments, Trenton, NJ) with a 10× objective lens. An 82000 optical filter cube (Chroma Technology Corp., Brattleboro, VT) was used for the excitation of calcein and CMTMR. Three images per well were collected and then analyzed using Metamorph software (Universal Imaging, West Chester, PA) for dye transfer from the donor to the acceptor cell. The scoring of fusion events was conducted as previously described [18]. The results were normalized by the control fusion and expressed as a percentage. Curves for fusion inhibition assays were fitted, using a hyperbolic decay function, and the IC50 was extracted. Fusion kinetics curves were fit to a sigmoidal function and the half time, at which 50% fusion was achieved, was extracted.

### Env:Receptor binding assay

gp120s were produced from 293T cells calcium phosphate transfected with gp120 expression constructs and infected with vTF1.1 vaccinia virus. Cell culture supernatants were harvested 24 hours post transfection/infection and gp120 concentrations determined by ELISA as previously described [42] with the exception that different antibodies were utilized. Receptor binding efficiencies of gp120s were determined using a cell surface-binding assay in which bound protein is detected by Western blot analysis [38,43]. Briefly, 2 × 10^6 ^QT6 target cells were calcium phosphate transfected with 6 ug pcDNA3.1 (control), CD4 or CXCR4 expression plasmids in 25 cm^2 ^culture flasks and infected with vTF1.1 to boost expression. 24 hrs post-transfection/infection, cells were incubated with gp120 for 2 hours at room temperature with and without 5 ug/ml soluble CD4 (sCD4) to trigger coreceptor binding site exposure. Cells were washed 3× with cold PBS to remove unbound gp120 then lysed with NP40 lysis buffer (0.5% NP40, 150 mM NaCl, 50 mM Tris pH 8) on ice for 10 minutes. Clarified lysates were assayed for gp120 content by SDS-PAGE and Western blotting and detected with an HIV-1 Env specific rabbit serum and an HRP-conjugated anti-rabbit antibody (Amersham Life Science, Piscataway, NJ) or a HIV-2 Env reactive MAb and an HRP-conjugated anti-mouse antibody (Promega, Madison, WI) followed by Supersignal chemiluminescent substrate (Pierce, Rockford, IL). Band intensity was quantitated using Kodak 1D software.

## Competing interests

The author(s) declare that they have no competing interests.

## Authors' contributions

SAG and HG performed the kinetic studies of HIV env-mediated fusion, JDR cloned and expressed HIV gp120 and performed the binding studies, BF performed the sequence alignment analysis, RWD participated in the design of the study and helped to draft the manuscript and RB conceived of the study, participated in its design and coordination and wrote the manuscript. All authors read and approved the final manuscript.

## References

[B1] Hahn BH, Shaw GM, De Cock KM, Sharp PM (2000). AIDS as a zoonosis: scientific and public health implications. Science.

[B2] Reeves JD, Doms RW (2002). Human immunodeficiency virus type 2. J Gen Virol.

[B3] Gallo SA, Sackett K, Rawat SS, Shai Y, Blumenthal R (2004). The Stability of the Intact Envelope Glycoproteins is a Major Determinant of Sensitivity of HIV/SIV to Peptidic Fusion Inhibitors. J Mol Biol.

[B4] Blumenthal R, Clague MJ, Durell SR, Epand RM (2003). Membrane fusion. Chem Rev.

[B5] Gustchina E, Hummer G, Bewley CA, Clore GM (2005). Differential inhibition of HIV-1 and SIV envelope-mediated cell fusion by C34 peptides derived from the C-terminal heptad repeat of gp41 from diverse strains of HIV-1, HIV-2, and SIV. J Med Chem.

[B6] Jernigan KM, Blumenthal R, Puri A (2000). Varying effects of temperature, Ca(2+) and cytochalasin on fusion activity mediated by human immunodeficiency virus type 1 and type 2 glycoproteins. FEBS Lett.

[B7] Puri A, Hug P, Munoz-Barroso I, Blumenthal R (1998). Human erythrocyte glycolipids promote HIV-1 envelope glycoprotein-mediated fusion of CD4+ cells. Biochem Biophys Res Commun.

[B8] Gallo SA, Finnegan CM, Viard M, Raviv Y, Dimitrov A, Rawat SS, Puri A, Durell S, Blumenthal R (2003). The HIV Env-mediated fusion reaction. Biochim Biophys Acta.

[B9] Reeves JD, Gallo SA, Ahmad N, Miamidian JL, Harvey PE, Sharron M, Pohlmann S, Sfakianos JN, Derdeyn CA, Blumenthal R, Hunter E, Doms RW (2002). Sensitivity of HIV-1 to entry inhibitors correlates with envelope/coreceptor affinity, receptor density, and fusion kinetics. Proc Natl Acad Sci U S A.

[B10] Reeves JD, Miamidian JL, Biscone MJ, Lee FH, Ahmad N, Pierson TC, Doms RW (2004). Impact of mutations in the coreceptor binding site on human immunodeficiency virus type 1 fusion, infection, and entry inhibitor sensitivity. J Virol.

[B11] Gallo SA, Puri A, Blumenthal R (2001). HIV-1 gp41 Six-Helix Bundle Formation Occurs Rapidly after the Engagement of gp120 by CXCR4 in the HIV-1 Env-Mediated Fusion Process. Biochemistry.

[B12] Chan DC, Kim PS (1998). HIV entry and its inhibition. Cell.

[B13] Furuta RA, Wild CT, Weng Y, Weiss CD (1998). Capture of an early fusion-active conformation of HIV-1 gp41. Nat Struct Biol.

[B14] Weissenhorn W, Dessen A, Calder LJ, Harrison SC, Skehel JJ, Wiley DC (1999). Structural basis for membrane fusion by enveloped viruses. Mol Membr Biol.

[B15] Doms RW, Moore JP (2000). HIV-1 membrane fusion: targets of opportunity. J Cell Biol.

[B16] Sodroski JG (1999). HIV-1 entry inhibitors in the side pocket. Cell.

[B17] Melikyan GB, Markosyan RM, Hemmati H, Delmedico MK, Lambert DM, Cohen FS (2000). Evidence that the transition of HIV-1 gp41 into a six-helix bundle, not the bundle configuration, induces membrane fusion. J Cell Biol.

[B18] Munoz-Barroso I, Durell S, Sakaguchi K, Appella E, Blumenthal R (1998). Dilation of the human immunodeficiency virus-1 envelope glycoprotein fusion pore revealed by the inhibitory action of a synthetic peptide from gp41. J Cell Biol.

[B19] Salzwedel K, Berger EA (2000). Cooperative subunit interactions within the oligomeric envelope glycoprotein of HIV-1: Functional complementation of specific defects in gp120 and gp41. Proceedings of the National Academy of Sciences.

[B20] Wyss S, Dimitrov AS, Baribaud F, Edwards TG, Blumenthal R, Hoxie JA (2005). Regulation of Human Immunodeficiency Virus Type 1 Envelope Glycoprotein Fusion by a Membrane-Interactive Domain in the gp41 Cytoplasmic Tail. J Virol.

[B21] Kim JT, Kim EM, Lee KH, Choi JE, Jhun BH, Kim JW (2002). Leucine zipper domain of HIV-1 gp41 interacted specifically with alpha-catenin. Biochem Biophys Res Commun.

[B22] Tencza SB, Miller MA, Islam K, Mietzner TA, Montelaro RC (1995). Effect of amino acid substitutions on calmodulin binding and cytolytic properties of the LLP-1 peptide segment of human immunodeficiency virus type 1 transmembrane protein. J Virol.

[B23] Zhang H, Wang L, Kao S, Whitehead IP, Hart MJ, Liu B, Duus K, Burridge K, Der CJ, Su L (1999). Functional interaction between the cytoplasmic leucine-zipper domain of HIV-1 gp41 and p115-RhoGEF. Curr Biol.

[B24] Evans DT, Tillman KC, Desrosiers RC (2002). Envelope glycoprotein cytoplasmic domains from diverse lentiviruses interact with the prenylated Rab acceptor. J Virol.

[B25] Wyss S, Berlioz-Torrent C, Boge M, Blot G, Honing S, Benarous R, Thali M (2001). The highly conserved C-terminal dileucine motif in the cytosolic domain of the human immunodeficiency virus type 1 envelope glycoprotein is critical for its association with the AP-1 clathrin adaptor [correction of adapter]. J Virol.

[B26] Miller MA, Cloyd MW, Liebmann J, Rinaldo CR, Islam KR, Wang SZ, Mietzner TA, Montelaro RC (1993). Alterations in cell membrane permeability by the lentivirus lytic peptide (LLP-1) of HIV-1 transmembrane protein. Virology.

[B27] Kliger Y, Shai Y (1997). A leucine zipper-like sequence from the cytoplasmic tail of the HIV-1 envelope glycoprotein binds and perturbs lipid bilayers. Biochemistry.

[B28] Comardelle AM, Norris CH, Plymale DR, Gatti PJ, Choi B, Fermin CD, Haislip AM, Tencza SB, Mietzner TA, Montelaro RC, Garry RF (1997). A synthetic peptide corresponding to the carboxy terminus of human immunodeficiency virus type 1 transmembrane glycoprotein induces alterations in the ionic permeability of Xenopus laevis oocytes. AIDS Res Hum Retroviruses.

[B29] Freed EO, Martin MA (1996). Domains of the human immunodeficiency virus type 1 matrix and gp41 cytoplasmic tail required for envelope incorporation into virions. J Virol.

[B30] Wyma DJ, Kotov A, Aiken C (2000). Evidence for a stable interaction of gp41 with Pr55(Gag) in immature human immunodeficiency virus type 1 particles. J Virol.

[B31] Wyma DJ, Jiang J, Shi J, Zhou J, Lineberger JE, Miller MD, Aiken C (2004). Coupling of human immunodeficiency virus type 1 fusion to virion maturation: a novel role of the gp41 cytoplasmic tail. J Virol.

[B32] Murakami T, Ablan S, Freed EO, Tanaka Y (2004). Regulation of human immunodeficiency virus type 1 Env-mediated membrane fusion by viral protease activity. J Virol.

[B33] Abrahamyan LG, Mkrtchyan SR, Binley J, Lu M, Melikyan GB, Cohen FS (2005). The cytoplasmic tail slows the folding of human immunodeficiency virus type 1 Env from a late prebundle configuration into the six-helix bundle. J Virol.

[B34] Edwards TG, Wyss S, Reeves JD, Zolla-Pazner S, Hoxie JA, Doms RW, Baribaud F (2002). Truncation of the cytoplasmic domain induces exposure of conserved regions in the ectodomain of human immunodeficiency virus type 1 envelope protein. J Virol.

[B35] Schols D, Struyf S, Van Damme J, Este JA, Henson G, De Clercq E (1997). Inhibition of T-tropic HIV strains by selective antagonization of the chemokine receptor CXCR4. J Exp Med.

[B36] Sattentau QJ, Dalgleish AG, Weiss RA, Beverley PC (1986). Epitopes of the CD4 antigen and HIV infection. Science.

[B37] Hoffman TL, LaBranche CC, Zhang W, Canziani G, Robinson J, Chaiken I, Hoxie JA, Doms RW (1999). Stable exposure of the coreceptor-binding site in a CD4-independent HIV- 1 envelope protein. Proc Natl Acad Sci U S A.

[B38] Lin G, Baribaud F, Romano J, Doms RW, Hoxie JA (2003). Identification of gp120 binding sites on CXCR4 by using CD4-independent human immunodeficiency virus type 2 Env proteins. J Virol.

[B39] Earl PL, Koenig S, Moss B (1991). Biological and immunological properties of human immunodeficiency virus type 1 envelope glycoprotein: analysis of proteins with truncations and deletions expressed by recombinant vaccinia viruses. J Virol.

[B40] Mulligan MJ, Ritter GD, Chaikin MA, Yamshchikov GV, Kumar P, Hahn BH, Sweet RW, Compans RW (1992). Human immunodeficiency virus type 2 envelope glycoprotein: differential CD4 interactions of soluble gp120 versus the assembled envelope complex. Virology.

[B41] Chakrabarti S, Mizukami T, Franchini G, Moss B (1990). Synthesis, oligomerization, and biological activity of the human immunodeficiency virus type 2 envelope glycoprotein expressed by a recombinant vaccinia virus. Virology.

[B42] Reeves JD, Schulz TF (1997). The CD4-independent tropism of human immunodeficiency virus type 2 involves several regions of the envelope protein and correlates with a reduced activation threshold for envelope-mediated fusion. J Virol.

[B43] Edinger AL, Blanpain C, Kunstman KJ, Wolinsky SM, Parmentier M, Doms RW (1999). Functional dissection of CCR5 coreceptor function through the use of CD4-independent simian immunodeficiency virus strains. J Virol.

